# Extraction of Balsam of Peru

**Published:** 1852-02

**Authors:** 


*Extraction of Balsam of Peru.*—It appears that there is an error in the account given in the works on Materia Medica, of the mode of preparing *Balsamum Peruvianum* (*Balsam of Peru,*) in Central America. In the last edition of the U. S. Dispensatory, the following description of the process is furnished: "Incisions are made into the bark, which is slightly burned, so as to cause the juice to flow. Cotton or woollen rags are then inserted into the apertures, and after saturation, are removed and replaced by others. When a sufficient quantity is collected, the rags are boiled in water in large jars; and the balsam rising to the surface, is skimmed off, and put in calabashes or bladders." The balsam, however, has a specific gravity of from 1.50 to 1.60, and cannot therefore, float in the water by boiling. This mistake was lately pointed out to Dr. Pereira, (who had adopted the erroneous description,) by Dr. Martius, of Erlangen; and Dr. Pereira, admitting the mis-statement, publishes in the London Pharmaceutical Journal, for November, 1851, the following paper on the mode of producing the balsam, furnished him, by a gentleman from South America, who obtained it "on the dictation of a native Indian."

"The manner used to extract the balsam from the trees, is to make several incisions in the tree, over which they place pieces of old cloth or rags, which absorb the juice, which, when they observe to be well soaked, they put in water to boil, until the rags have discharged the greatest part of the balsam which they had imbibed. They allow it to settle sufficiently until the water rises, leaving the balsam at the bottom; they pour off with care the water, afterwards filling '*tecomates*' (gourds,) with the balsam, although it is not now very pure. The rags are then put into 'redes' (little bags of cord,) which, when strongly twisted, wring any remaining balsam out of them into the 'tecomates.' When we purchase it, it is necessary to clean it again, because it still contains water and other impurities, which some Indians will mix with it to gain greater weight."

